# Pulmonary nocardiosis caused by *Nocardia cyriacigeorgica* in patients with *Mycobacterium avium*complex lung disease: two case reports

**DOI:** 10.1186/s12879-014-0684-z

**Published:** 2014-12-10

**Authors:** Kazuma Yagi, Makoto Ishii, Ho Namkoong, Takahiro Asami, Hiroshi Fujiwara, Tomoyasu Nishimura, Fumitake Saito, Yoshifumi Kimizuka, Takanori Asakura, Shoji Suzuki, Tetsuro Kamo, Sadatomo Tasaka, Tohru Gonoi, Katsuhiko Kamei, Tomoko Betsuyaku, Naoki Hasegawa

**Affiliations:** Department of Medicine, Division of Pulmonary Medicine, Keio University School of Medicine, 35 Shinanomachi, Shinjuku-ku, Tokyo, 160-8582 Japan; Center for Infectious Diseases and Infection Control, Keio University School of Medicine, 35 Shinanomachi, Shinjuku-ku, Tokyo, 160-8582 Japan; Keio University Health Center, 35 Shinanomachi, Shinjuku-ku, Tokyo, 160-8582 Japan; Division of Clinical Research, Medical Mycology Research Center, Chiba University, Inohana 1-8-1, Chuo-ku, Chiba, Japan; Division of Control and Treatment of Infectious Diseases, Chiba University Hospital, Inohana 1-8-1, Chuo-ku, Chiba, Japan

**Keywords:** Pulmonary nocardiosis, Mycobacterium avium complex lung disease, Nocardia cyriacigeorgica

## Abstract

**Background:**

Pulmonary nocardiosis frequently occurs in immunocompromised hosts and in some immunocompetent hosts with chronic lung disease; however, few reports have described pulmonary nocardiosis with nontuberculous mycobacterial lung infection. Here we report for the first time two cases of pulmonary nocardiosis caused by *Nocardia cyriacigeorgica* associated with *Mycobacterium avium* complex (MAC) lung disease caused by *M. avium*.

**Case presentation:**

Case 1 is that of a 72-year-old Japanese man with untreated MAC lung disease, who was diagnosed with rheumatoid arthritis and initiated on methotrexate. After 3 years of methotrexate therapy, the patient remained smear-negative and culture-positive for MAC, but also became smear-positive for *Nocardia* species. He received trimethoprim/sulfamethoxazole, and his symptoms and lung infiltrates improved. Case 2 is that of an immunocompetent 53-year-old Japanese woman with MAC lung disease, who was treated with a combined therapy of clarithromycin, rifampicin, ethambutol, and levofloxacin. MAC sputum culture was negative after 1 year of combined treatment, which was maintained for 2 years. After four treatment-free years, *Nocardia* species were occasionally isolated from her sputum, although MAC was rarely isolated from sputum cultures over the same period. In both cases, the *Nocardia* species were identified as the recently defined *N. cyriacigeorgica* by 16S ribosomal RNA gene sequencing.

**Conclusion:**

We report two cases of pulmonary nocardiosis caused by *N. cyriacigeorgica* associated with MAC lung disease caused by *M. avium* and suggest that *N. cyriacigeorgica* may be a major infective agent associated with MAC lung disease.

**Electronic supplementary material:**

The online version of this article (doi:10.1186/s12879-014-0684-z) contains supplementary material, which is available to authorized users.

## Background

Pulmonary nocardiosis is an uncommon pulmonary infection caused by aerobic gram-positive actinomycetes of the genus *Nocardia* [1]. It occurs mainly as an opportunistic infection in immunocompromised patients, particularly in those with defects in cell-mediated immunity such as patients with human immunodeficiency virus (HIV) infection and those receiving long-term systemic steroids or immunosuppressive agents; however, it can also affect immunocompetent hosts with no underlying disease [2]-[6]. Pulmonary nocardiosis often occurs with chronic lung diseases such as chronic obstructive pulmonary disease (COPD) and bronchiectasis [2]-[4].

*Mycobacterium avium* complex (MAC) lung disease is becoming more prevalent [7]. It often occurs in immunocompromised hosts such as patients with HIV and in immunocompetent hosts with chronic lung diseases such as COPD and bronchiectasis; however, around 20% of MAC patients have no underlying disease [8]. A subset of patients with MAC lung disease may also experience pulmonary nocardiosis. Previous reports have described a case of pulmonary *Nocardia farcinica* infection in a patient with *Mycobacterium intracellulare* infection and bronchiectasis [9] and a case of pulmonary *Nocardia asteroides* infection in a patient with MAC infection, following allogeneic bone marrow transplantation [10]. Co-infection with *N. asteroides* and MAC has also been reported in a patient with AIDS [11]. Another report described a case of empyema caused by *Mycobacterium tuberculosis*, *Nontuberculous mycobacteria* (species not identified), and *N. asteroides* isolated from pleural effusion in a middle-aged woman with systemic lupus erythematosus, treated with long-term corticosteroids [12]. There has been no similar report of pulmonary nocardiosis caused by *Nocardia cyriacigeorgica* in patients with MAC lung disease.

To the best of our knowledge, this is the first report of two cases of pulmonary *N. cyriacigeorgica* infection with MAC lung disease caused by *M. avium*, including an immunocompromised 72-year-old man with rheumatoid arthritis (RA), treated with methotrexate for several years (Case 1), and an immunocompetent 53-year-old woman (Case 2).

## Case presentation

**Figure 1:**
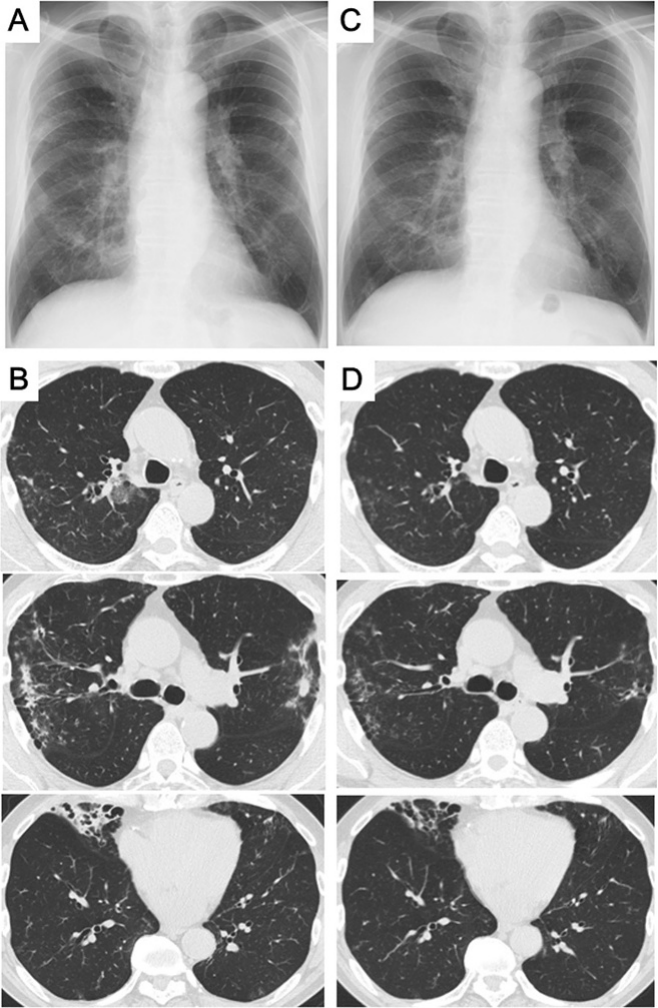
**Chest X-ray and chest computed tomography (CT) findings in Case 1.** Chest X-ray and chest CT findings examined at the time of therapeutic initiation with trimethoprim-sulfamethoxazole **(A and** **B)** and after three months of therapy **(C and** **D)**.

Case 1 is that of a 72-year-old Japanese man with untreated MAC lung disease caused by *M. avium*, which was followed 2 years later with a clinical diagnosis of RA; he was referred to a university hospital in 2009. Treatment with methotrexate was initiated for high RA disease activity. Although MAC was continuously isolated from his sputum, he was carefully followed without medical treatment because smear tests were negative, he had no respiratory symptoms, and lung infiltrates were stable. After 3 years of methotrexate therapy, *Nocardia* species appeared and were continuously isolated from his sputum, which was smear-negative but culture-positive for MAC. His sputum amount increased and chest X-ray imaging and computed tomography (CT) revealed exacerbation of infiltrates in the upper lobes on both sides and in the right middle lobe of the lungs (Figure 1A and B). He had no smoking history and neither cutaneous nor central nervous system nocardiosis. His laboratory findings were almost normal, including normal interferon-gamma release assays (IGRA), with the exception of a high titer of anti-tuberculous glycolipid (TBGL) antigen antibody (19.0 U/mL) and anti-glycopeptidolipid (GPL) core antigen immunoglobulin A (IgA) antibody (4.6 U/mL). He started receiving treatment with trimethoprim/sulfamethoxazole (TMP/SMX) in August 2012. His symptoms and the infiltrates improved after 3 months of therapy (Figure 1C and D).

**Figure 2:**
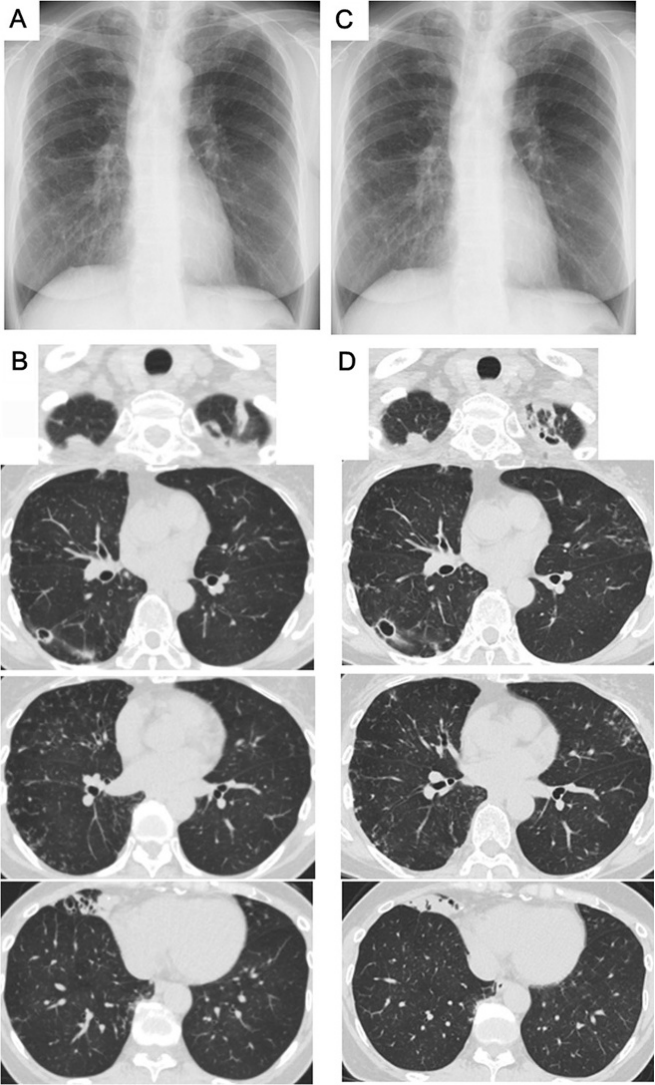
**Chest X-ray and chest computed tomography (CT) findings in Case 2.** Chest X-ray and chest CT findings 15 months before the exacerbation of lung infiltrates **(A and** **B)** and at the time of exacerbation **(C and** **D)**.

Case 2 is that of a 53-year-old Japanese woman referred to a university hospital as an outpatient because of an abnormal shadow upon chest X-ray imaging in 2001. She was a former smoker with a 6-pack-year smoking history (4 cigarettes per day for 30 years). The forced expiratory volume in 1 second/forced vital capacity (FEV_1_/FVC) ratio was slightly decreased (68.7%). Since her sputum smear and culture were positive for MAC and chest CT revealed widespread bilateral nodular bronchiectatic pulmonary infiltrates, she was initiated on combined therapy with clarithromycin (400 mg/day), rifampicin (600 mg/day), ethambutol (1000 mg/day), and levofloxacin (400 mg/day) shortly after diagnosis in 2001. The sputum MAC culture turned negative 1 year after initiating therapy and she completed 2 years of treatment in 2003. After four treatment-free years, *Nocardia* species appeared in her sputum in 2007. We continued careful follow-up without medical treatment because she was immunocompetent and showed no symptoms except for a very mild cough; her sputum culture was often negative for *Nocardia* species and lung infiltrates were stable. Chest X-ray imaging and CT in 2013 revealed an exacerbation of infiltrates in the right middle lobe, a small cavity in the right lower lobe, and centrilobular nodules in the left upper lobe of the lungs (Figure 2C and D) in comparison to images obtained 15 months before exacerbation of the infiltrates (Figure 2A and B). She was admitted to the University Hospital for bronchoscopy. Her physical examination was unremarkable and she had neither cutaneous nor central nervous system nocardiosis. Her laboratory findings were almost normal, including normal IGRA, except for a high titer of anti-TBGL antigen antibody (9.9 U/mL) and anti-GPL core antigen IgA antibody (>10.00 U/mL). Bronchial washing, bronchial curettage, and transbronchial lung biopsy through right B^5^ and B^6^ were performed. *Nocardia* species but not MAC were cultured from samples of the bronchial wash. The pathogen responsible for the exacerbated infiltrates on chest CT was accordingly considered to be *Nocardia*. The patient is being followed closely; the decision to treat for *Nocardia* remains pending a change in her status.

In both cases, *Nocardia* specie*s* isolated from sputum were identified as *N. cyriacigeorgica* by 16S ribosomal RNA gene sequencing. Table 1 shows the minimum inhibitory concentration (MIC) of the indicated antibiotics against *N. cyriacigeorgica* in each case. Susceptibility testing was performed according to Clinical and Laboratory Standards Institute (CLSI) document M24-A [13]; the specific susceptible breakpoint of TMP/SMX for *Nocardia* species was ≤2/38 and the specific resistance breakpoint was ≥4/76. The MICs of TMP/SMX for *N. cyriacigeorgica* were 0.5/9.5 in Case 1 and 8/152 in Case 2. Thus, the *N. cyriacigeorgica* isolate in Case 1 was regarded as sensitive to TMP/SMX, while the isolate in Case 2 was regarded as resistant.

**Table 1:** **Antimicrobial susceptibilities of** ***Nocardia cyriacigeorgica*** **isolated from sputum cultures**

	MIC in case 1 (μg/mL)	MIC in case 2 (μg/mL)
Ampicillin	>8	>8
Cefotaxime	>64	8
Imipenem	1	1
Minocycline	<0.25	1
Trimethoprim/Sulfamethoxazole	0.5/9.5	8/152
Amikacin	4	1
Gentamicin	<0.5	<0.5
Ciprofloxacin	4	>4
Clarithromycin	2	>8
Erythromycin	>2	>2

*MIC* minimum inhibitory concentration.

### Discussion

Pulmonary nocardiosis is the most frequent presentation of infection by *Nocardia* species [1], associated with opportunistic infections in immunocompromised hosts, particularly in those with impaired cell-mediated immunity, although immunocompetent hosts can also present with pulmonary nocardiosis. In spite of the fact that *Nocardia* infection is relatively rare, early diagnosis and treatment is important because of its poor prognosis in selected cases. A few reports have described pulmonary nocardiosis in patients with nontuberculous mycobacterial lung infection [9]-[12]. Here, we report two cases of pulmonary *N. cyriacigeorgica* infection in patients with MAC lung disease.

Accumulating evidence suggests there are defined risk factors for *Nocardia* species infection: HIV infection [5], corticosteroid therapy or use of immunosuppressive agents [5],[6], organ transplantation [6], and diabetes mellitus [3]. Preexisting rheumatic disease treated with immunosuppressant therapy, as in Case 1, is another risk factor for *Nocardia* species infection [14]. Preexisting pulmonary diseases such as COPD and bronchiectasis are additional risk factors for pulmonary nocardiosis, which occur when bacterial colonization in the lower respiratory tract alters ciliary motility and causes epithelial damage [4]. It is unclear whether preexisting nontuberculous mycobacterium is an independent risk factor for pulmonary nocardiosis, although colonization in the lower respiratory tract may also alter ciliary motility and cause epithelial damage as well as COPD and bronchiectasis. Intrinsic defects in airway clearance may also contribute to the susceptibility to pulmonary nontuberculous mycobacterial infection and nocardiosis. Indeed, a previous report demonstrated that mutations in the cystic fibrosis transmembrane regulator likely contribute to susceptibility and pathogenesis in adults with bronchiectasis and pulmonary nontuberculous mycobacterial infection [15]. Airway clearance therapies are likely to function in synergy with antimicrobial therapy to accelerate resolution of airway infections. In Case 2, the patient had a long-term history of smoking (4 cigarettes per day for 30 years) and the FEV_1_/FVC ratio was slightly reduced at the initial visit. Although chest CT did not show pulmonary emphysema, she may have a chronic bronchitis form of mild COPD and mild bronchiectasis. Preexisting COPD, bronchiectasis, and MAC lung disease may contribute to the co-infection with *Nocardia* in Case 2.

The *Nocardia* species isolated from our patients was *N. cyriacigeorgica*, a recently identified species confirmed by 16S ribosomal RNA gene sequencing. *N. cyriacigeorgica* was first classified in 2001 by Yassin et al. as a novel species distinguished by molecular and biochemical analysis from previously identified members of the genus *Nocardia* such as *N. asteroides* [16]. A recent study showed that a quarter of the strains from Japan and Thailand that were identified as *N. asteroides* in fact belonged to *N. cyriacigeorgica* (27 of 121 cases) [17]. In addition, *N. cyriacigeorgica* is the most frequently isolated strain in Taiwanese patients with pulmonary nocardiosis (10 of 20 cases) [18]. *N. cyriacigeorgica* infection may be most common in East and Southeast Asia. Larger populations should be studied to improve our understanding of the characteristics of pulmonary *N. cyriacigeorgica* infection.

*Nocardia* species co-infection may be underestimated in patients with MAC lung disease because *Nocardia* and MAC infection often develop in immunocompromised hosts and in selected immunocompetent hosts with chronic lung disease. Chest CT findings in MAC lung disease, such as nodular bronchiectatic lesions, often resemble those in *Nocardia* species infection; thus, clinicians may not suspect the relatively rare *Nocardia* species co-infection. *Nocardia* species also grow slowly and positive cultures may take up to 4 weeks to appear; thus, clinicians may not obtain positive culture results for *Nocardia* species unless they proactively suspect *Nocardia* infection and incubate the samples for longer than normal.

TMP/SMX is widely used in first-line therapy of pulmonary nocardiosis. Amikacin, imipenem, third-generation cephalosporins, minocycline, netilmicin, and amoxicillin-clavulanic acid are also effective against *Nocardia* isolates *in vitro* and can be used as alternative antimicrobial agents [4]. However, no randomized prospective controlled trials have been performed to determine the most appropriate therapeutic agent, route of administration, and treatment duration for patients with pulmonary nocardiosis [2]. *N. cyriacigeorgica* isolated in Case 2 was resistant to TMP/SMX, according to the CLSI definition of susceptibility. TMP/SMX may be excluded as a candidate drug for treatment of pulmonary nocardiosis in Case 2. However, susceptibility test results do not always correlate with clinical outcomes of treatment for *Nocardia* [19]. In fact, a favorable response was reported in 3 of 4 cases (75%) with TMP/SMX-resistant strains [19]. Therefore, TMP/SMX remains a candidate antimicrobial agent to treat pulmonary nocardiosis in Case 2.

## Conclusions

This is the first report of pulmonary nocardiosis due to *N. cyriacigeorgica* in patients with MAC lung disease. *Nocardia* species co-infection may be underestimated in patients with MAC lung disease and *N. cyriacigeorgica* may be the main *Nocardia* species co-incident with MAC lung disease.

## Consent

Written informed consent was obtained from each patient for publication of this Case report. Copies of the written consents are available for review by the Editor of this journal.

## Authors’ original submitted files for images

Below are the links to the authors’ original submitted files for images. Authors’ original file for figure 1 Authors’ original file for figure 2

## Abbreviations

HIV: Human immunodeficiency virus
COPD: Chronic obstructive pulmonary disease
MAC: *Mycobacterium avium* complex
RA: Rheumatoid arthritis
CT: Computed tomography
IGRA: Interferon-gamma release assays
TBGL: Tuberculous glycolipid
GPL: Glycopeptidolipid
IgA: Immunoglobulin A
TMP/SMX: Trimethoprim/sulfamethoxazole
FEV_1_/FVC: Forced expiratory volume in 1 second/forced vital capacity
MIC: Minimum inhibitory concentration
CLSI: Clinical and Laboratory Standards Institute
