# Supplementary figures and images for: Pulmonary nocardiosis caused by Nocardia cyriacigeorgica in patients with Mycobacterium aviumcomplex lung disease: two case reports
Source: BMC Infect Dis. 2014 Dec 10;14:684. doi: 10.1186/s12879-014-0684-z (PMC4266951; doi:10.1186/s12879-014-0684-z)

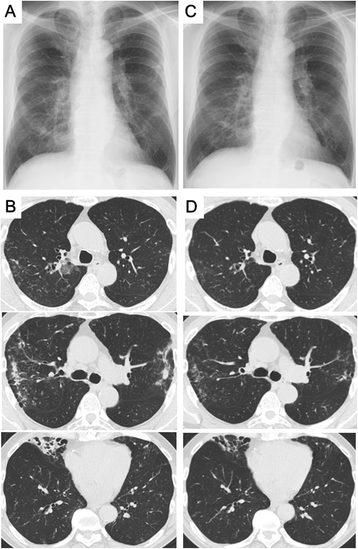

Supplement: Supplementary file 1 — Authors’ original file for figure 1 [file 12879_2014_684_MOESM1_ESM.gif]

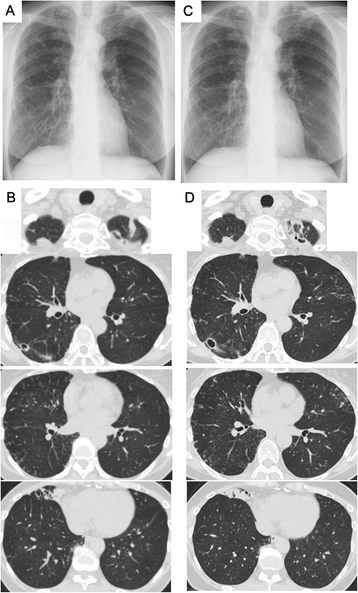

Supplement: Supplementary file 2 — Authors’ original file for figure 2 [file 12879_2014_684_MOESM2_ESM.gif]
